# Paroxysmal Sympathetic Hyperactivity in a Young Male with Glioblastoma Multiforme

**DOI:** 10.7759/cureus.6933

**Published:** 2020-02-10

**Authors:** Mohamed S Suliman, Varun Dobariya, Mena Shehata, Davinder Singh, Amro Al-Astal

**Affiliations:** 1 Internal Medicine, Marshall University, Joan C. Edwards School of Medicine, Huntington, USA; 2 Internal Medicine: Pulmonology, Marshall University, Joan C. Edwards School of Medicine, Huntington, USA

**Keywords:** acquired brain injury, glioblastoma multiforme, paroxysmal sympathetic hyperactivity, dysautonomia, sympathetic storm, paroxysmal autonomic instability with dystonia

## Abstract

Paroxysmal sympathetic hyperactivity (PSH) is a rare syndrome that is a recognized complication of severe brain injury. It is characterized by episodic hypertension, hyperthermia, tachycardia, tachypnea, diaphoresis, and specific posturing. It is prevalent in an intensive care unit (ICU) setting where high acuity illnesses can mask the symptoms of PSH. Herein, we report a case of PSH in a patient with a past medical history significant for glioblastoma multiforme (GBM) status post hemicraniectomy, radiation, and chemotherapy.

## Introduction

It is common for physicians who treat patients with traumatic brain injuries to see wide fluctuations in heart rate, respiratory rate, and blood pressure. For decades, these vital sign fluctuations were hypothesized to stem from increased pressure on the thalamus. Originally termed diencephalic autonomic seizures by Dr. Wilder Penfield in 1929, paroxysmal sympathetic hyperactivity (PSH) is now known to be a well-recognized syndrome as a result of severe brain injury [1-2]. PSH is characterized by episodic hypertension, hyperthermia, tachycardia, tachypnea, diaphoresis, and extensor posturing. The mechanism of PSH has been described as autonomic dysfunction in the diencephalon or its connections but it has not been fully elucidated [3]. There is a lack of clear terminology and definition, which probably is a cause and consequence of the under-recognition of this syndrome, despite its relatively high incidence after severe brain damage. It can result in increased morbidity and is associated with a higher cost of care. The pathophysiology is complex and is further compounded by a failure to distinguish between mixed parasympathetic/sympathetic and pure sympathetic hyperactivity, with a conflation of both in a single diagnosis for many decades [4].

## Case presentation

**Figure 1:**
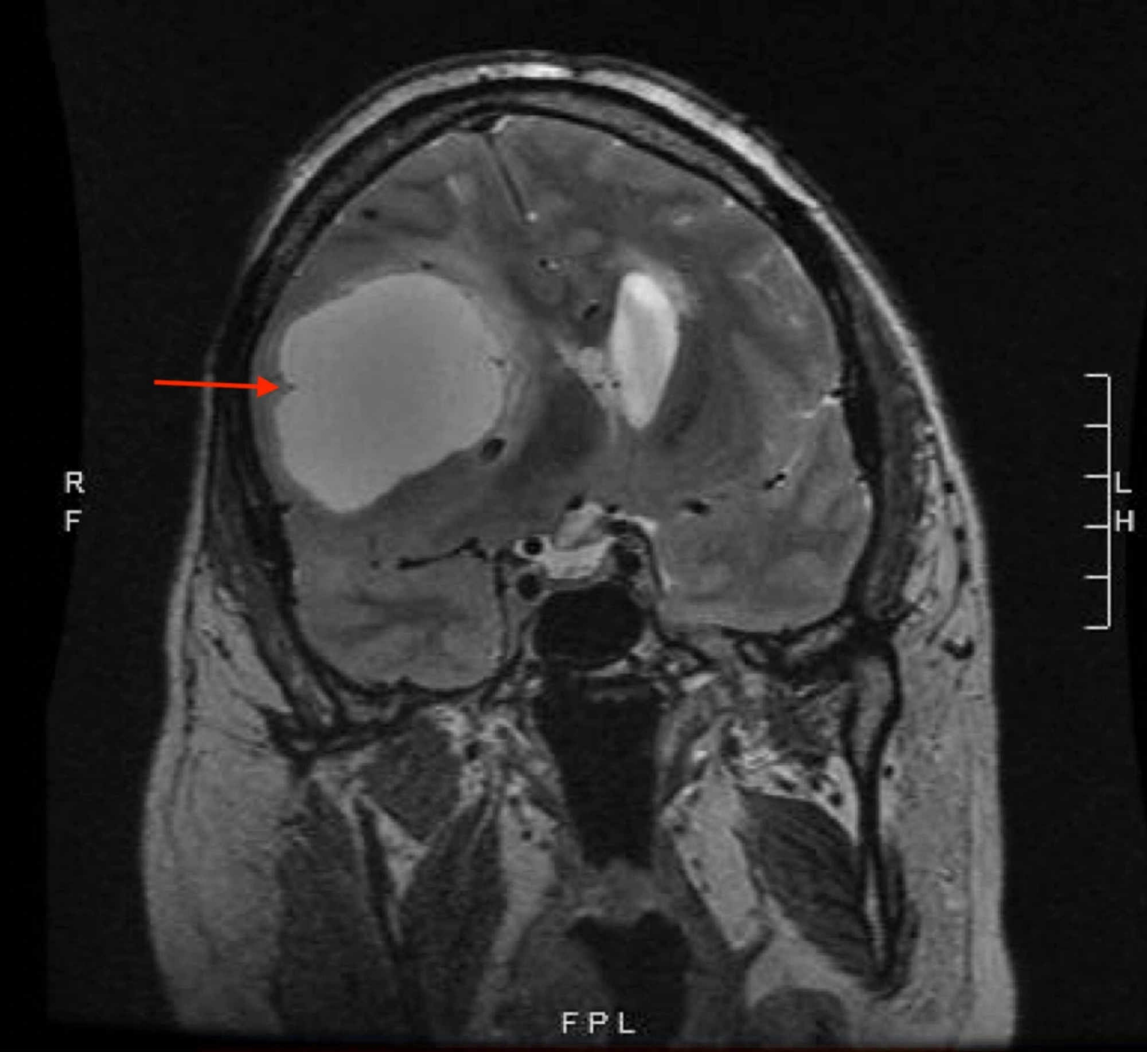
Large multilobulated right soft tissue mass

A 44-year-old male patient, with a past medical history significant for glioblastoma multiforme (GBM) status-post right hemicraniectomy seven years prior to presentation, was evaluated in the emergency department (ED) with episodic seizure-like activity, which was witnessed and reported by his wife as tonic flexion of his right arm and leg with gaze deviation and an altered mental status. Historically, he underwent radiation therapy to the tumor bed and was continued on temozolomide. Prior magnetic resonance imaging (MRI) showed disease progression and he was, therefore, continued on carbamazepine and levetiracetam for tumor-induced seizures. MRI upon presentation revealed significant cortical atrophy with ventriculomegaly and a large multilobulated soft tissue mass measuring 8.0x7.4 cm in the axial dimension; the latter remained unchanged when compared to prior MR images (Figure 1).

**Figure 2:**
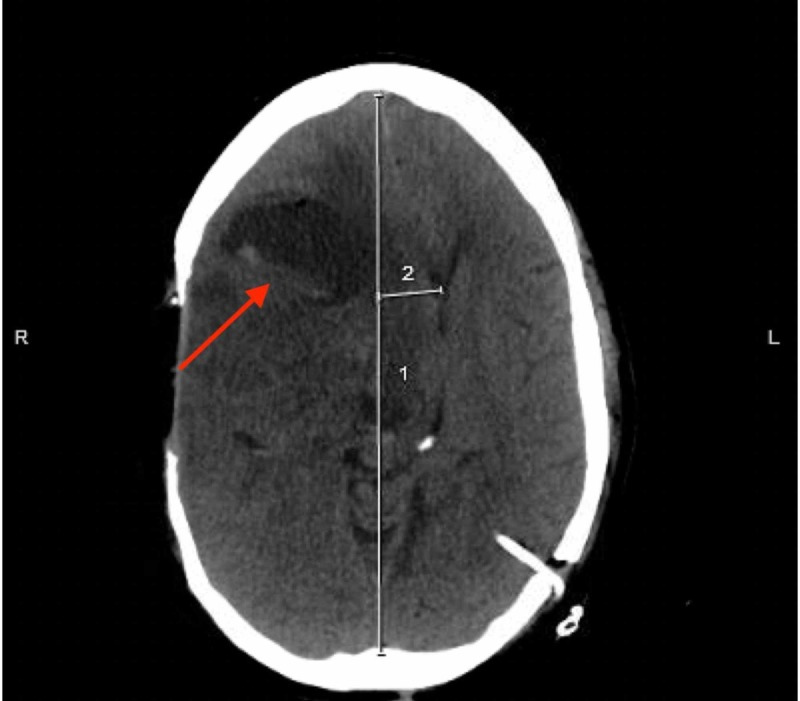
Worsening edema and progressive midline shift

The patient was admitted to the neurology floor and was started on intravenous levetiracetam. Continuous electroencephalogram (EEG) with video monitoring showed a severe generalized slowing in the theta range, with superimposed right frontal, temporal, and parietal sharp waves as well as polymorphic delta waves indicating focal cerebral dysfunction with potential epileptogenicity. During hospitalization, he developed episodic hypertension and tachycardia associated with his seizure-like activity and was managed with lorazepam injections with no response. He was transferred to the intensive care unit (ICU) for the management of status epilepticus and intubated for airway protection in the setting of altered mental status. The patient was sedated with midazolam and fentanyl. The patient continued manifesting episodes of tachypnea, tachycardia, and severe hypertension in association with dystonia. The neurology service evaluated the patient and the diagnosis of PSH was confirmed by exclusion. Intravenous labetalol and clonidine were administered, resulting in dramatic improvement with symptomatic control of his paroxysmal symptoms. Despite symptom improvement, repeat brain imaging with computed tomography (CT) revealed worsening edema and progressive midline shift (Figure 2).

The family was presented and educated with the option of palliative care; yet, they opted for neurosurgical shunt placement. Post-operatively, the patient developed a brain hemorrhage and hospice care was pursued.

## Discussion

PSH is a syndrome associated with trauma to the brain, stroke, encephalitis, and various other forms of brain injuries. In an effort to establish a strong correlation between PSH and traumatic brain injury (TBI), a review of 349 PSH case reports published before 2010 found that about 80% followed TBI, 10% followed anoxic brain injury, 5% followed stroke, and the remaining 5% were associated with hydrocephalus, tumors, infections, or unspecified causes [5]. Sympathetic hyperactivity is usually the hallmark of this syndrome, and 80% of cases occur secondary to traumatic brain injury. The most dramatic of the presentations in this syndrome include paroxysmal severe tachycardia, arterial hypertension, tachypnea, hyperthermia, and decerebrate posturing [4].

PSH has been reported in conditions other than TBI. These include hypoxic brain injuries, brain tumors, hydrocephalus, and subarachnoid hemorrhages [2]. Although the pathophysiology of PSH is not known, it has been postulated as a result of the disinhibition of the central sympathoexcitatory regions, leading to catecholamine release and a subsequent hyperadrenergic state [6]. Several central neurotransmitter systems have been implicated in the maladaptive responses that drive PSH. Regardless of these central neurotransmitter changes, good evidence supports an association between PSH and peripheral catecholamines, and possibly corticosteroid release, which might explain the exaggerated responses to non-noxious or mildly noxious stimuli observed in patients with PSH [4].

Differential diagnosis is often challenging, especially in the phase of concurrent brain pathology. A clue to the diagnosis is the paroxysmal nature of symptoms and signs of PSH followed by the return of vitals to baseline between episodes. Because patients may be heavily sedated for ventilation or pain management, temperature elevation is usually the first concern, prompting the need to rule out sepsis. Elevated blood pressure and dystonic features raise suspicion for intracranial abnormalities such as hydrocephalus, increased intracranial pressure, and blood or fluid accumulation. Treating patients with PSH following a brain injury is always a difficult task and remains controversial.

Numerous treatment and palliation options have been proposed using multistudy reviews of PSH. Since PSH is a syndrome of overexcited sympathetic tone, it is logical that effective treatment strategies would be by attempting to reduce or block sympathetic nervous system activity. Suggested medications include clonidine, dantrolene, lorazepam, morphine sulfate, and nonselective beta-blockers. These may be used individually or in combination [2]. In our patient, a symptom triggered approach was pursued once the diagnosis was established and, eventually, palliation was decided upon by the family. A reasonable approach is to choose target signs, consider the safety of particular drugs for the individual, and set a time frame to determine efficacy before a drug is changed or before the addition of a second agent. A more detailed understanding of PSH and earlier intervention may minimize cumbersome testing, avoid polypharmacy, and alleviate the anxiety of all those involved in caring for this group of patients.

## Conclusions

PSH is an important clinical problem in critically ill neurologic patients. It can lead to poor outcomes and a prolonged hospital stay. Recognizing PSH early and using a multidisciplinary approach involving neurology, surgery, and critical care is essential to decrease morbidity, cost of care, and length of stay.
